# The Associations Between Preoperative Anthropometry and Postoperative Outcomes in Infants Undergoing Congenital Heart Surgery

**DOI:** 10.3389/fcvm.2022.812680

**Published:** 2022-04-01

**Authors:** Jia Yi Joel Lim, Rui Wen Bryan Wee, Mihir Gandhi, Yee Phong Lim, Li Nien Michelle Tan, Swee Chye Quek, Marion M. Aw, Ching Kit Chen

**Affiliations:** ^1^Yong Loo Lin School of Medicine, National University of Singapore, Singapore, Singapore; ^2^Biostatistics, Singapore Clinical Research Institute, Consortium for Clinical Research and Innovation Singapore, Singapore, Singapore; ^3^Centre for Quantitative Medicine, Duke-NUS Medical School, Singapore, Singapore; ^4^Tampere Center for Child Health Research, Tampere University, Tampere, Finland; ^5^Department of Paediatrics, Yong Loo Lin School of Medicine, National University of Singapore, Singapore, Singapore; ^6^Division of Gastroenterology, Nutrition, Hepatology and Liver Transplantation, Department of Paediatrics, Khoo Teck Puat–National University Children's Medical Institute, National University Health System, Singapore, Singapore; ^7^Division of Cardiology, Department of Paediatrics, Khoo Teck Puat–National University Children's Medical Institute, National University Health System, Singapore, Singapore

**Keywords:** congenital heart disease, feeding difficulties, infants, cardiac surgery, nutritional status, preoperative weight-for age z-score, postoperative complications, 6-month mortality

## Abstract

**Aim:**

We explored the association between preoperative anthropometry and biochemistry, and postoperative outcomes in infants with CHD after cardiac surgery, as infants with congenital heart disease (CHD) often have feeding difficulties and malnutrition.

**Methodology:**

This was a retrospective review of infants (≤ 1-year-old) who underwent congenital heart surgery. Preoperative anthropometryin terms of preoperative weight-for-age z-score (WAZ), length-for-age z-score (LAZ), as well as preoperative serum albumin and hemoglobin concentrations, were evaluated against 6-month mortality, and morbidity outcomes including postoperative complications, vasoactive inotrope score, duration of mechanical ventilation, length of stay in the pediatric intensive care unit and in hospital, using the logistic regression or median regression models accounting for infant-level clustering.

**Results:**

One hundred and ninety-nine operations were performed in 167 infants. Mean gestational age at birth was 38.0 (SD 2.2) weeks (range 26 to 41 weeks). Thirty (18.0%) infants were born preterm (<37 weeks). The commonest acyanotic and cyanotic lesions were ventricular septal defect (26.3%, 44/167), and tetralogy of Fallot (13.8%, 23/167), respectively. Mean age at cardiac surgery was 94 (SD 95) days. Feeding difficulties, including increased work of breathing during feeding, diaphoresis, choking or coughing during feeding, and inability to complete feeds, was present in 54.3% (108/199) of infants prior to surgery, of which 21.6% (43/199) required tube feeding. The mean preoperative WAZ was−1.31 (SD 1.79). Logistic regression models showed that low preoperative WAZ was associated with increased risk of postoperative complications (odds ratio 1.82; *p* = 0.02), and 6-month mortality (odds ratio 2.38; *p* = 0.008) following CHD surgery. There was no meaningful association between the other preoperative variables and other outcomes.

**Conclusion:**

More than 50% of infants with CHD undergoing cardiac surgery within the first year of life have feeding difficulties, of which 22% require to be tube-fed. Low preoperative WAZ is associated with increased postoperative complications and 6-month mortality.

## Introduction

Congenital heart disease (CHD) is the most common birth defect in newborns, affecting >1 million live births per annum globally and causing 10% of stillbirths. The moderate and severe forms of CHD affect ~6–20 per thousand live-births (1), and is a major cause of infant mortality and morbidity in the developed world (2). The majority of infants with CHD have normal weight for gestational age at birth (3), but a significant proportion of them develop malnutrition and growth deficiencies in the first year of life (4–6).

While cardiac surgery in these infants reduces CHD-related mortality, and improves growth and development (7–9), malnutrition significantly militates against successful outcomes. Infants with CHD are at risk of malnutrition and growth failure due to both cardiac and extracardiac factors, including feeding difficulties, inadequate caloric intake, inefficient nutrient absorption and utilization, and increased metabolic demands (10, 11). The severity and type of malnutrition may be related to factors such as the presence of cyanosis, congestive cardiac failure, and pulmonary hypertension (5, 6, 12–14). To date, there are few studies that examine the combination of both anthropometric and biochemical markers when assessing preoperative nutrition in infants with CHD, particularly those requiring cardiac surgery in the first year of life. Furthermore, previous studies focused on short-term outcomes for CHD surgeries which are determined by surgical and intensive care expertise to a large extent, rather that patient-related factors.

Our study sought to evaluate the presence and the magnitude of feeding difficulties in infants with CHD in an Asian population, and to determine the impact of nutritional status, a potentially modifiable risk factor, on surgical outcomes for CHD infants undergoing cardiac surgery. This will be helpful in informing future decision-making regarding the use of anthropometric and biochemical markers to evaluate and optimize nutrition preoperatively, and hence enhance post-surgical outcomes both in the short- and long-term. This study is unique as it focuses specifically on infants undergoing CHD surgery, a specific population who may be particularly vulnerable to nutritional deficits during this period of rapid somatic growth.

## Materials and Methods

### Study Design

This was a single-center, retrospective cohort study that was approved by the institutional review board with a waiver of informed consent. Patients were identified through the institutional cardiac surgical database at the National University Hospital, Singapore. All index CHD surgeries in newborns and infants (age ≤ 1 year) between January 2014 and December 2018 were eligible for inclusion. Index surgery refers to the first surgery of the hospitalization. The analysis is based on unique hospitalization episodes, with patient characteristics evaluated at the time of the index surgery of the episode. Subsequent surgeries during the same hospitalization were not counted as separate cases i.e., each admission for surgery was considered separately for infants requiring multiple / staged operations. Newborns and infants with genetic syndromes such as trisomy 21 or DiGeorge syndrome (22q.11 microdeletion) were included, as we were interested in evaluating nutritional status of all infants considered to have increased nutrition risk. Nevertheless, we were cognizant of the limitations to this approach as growth in infants with CHD and genetic abnormalities may have different predicted growth trajectories.

### Data Collection

#### At Birth and Surgical Characteristics

Data were retrieved from inpatient and outpatient medical records. Demographic characteristics collected include age at surgery, sex, gestational age at birth, birth weight, birth length, and ethnicity. Clinical characteristics collected include the cardiac diagnosis (classified into cyanotic vs. acyanotic cardiac lesions), preoperative medications (e.g., diuretics for symptoms of congestive heart failure, prostaglandin to maintain patency of ductus arteriosus in duct-dependent circulations, etc.), priority of surgery (elective vs. emergency), and type of surgical procedure. The Risk Adjustment for Congenital Heart Surgery (RACHS)-1 risk categories were used to classify the complexity of the various surgical procedures and their associated mortality risk (15). Operative characteristics recorded were cardiopulmonary bypass (CPB) time, aortic cross-clamp (AXC) time, deep hypothermic circulatory arrest (DHCA) time, and delayed sternal closure.

#### Preoperative Characteristics

Preoperatively, recumbent length and weight were measured using a length mat, and an infant weighing scale, respectively. Length and weight z-scores for age (length-for-age z-score, LAZ; weight-for-age z-score, WAZ) were calculated using the World Health Organization (WHO) growth standards for ages 0 to 24 months (16, 17). Preoperative serum albumin and hemoglobin concentrations, which were measured before surgery as part of a routine preoperative assessment, were recorded. Nutritional status, as measured by WAZ, LAZ, serum albumin and hemoglobin concentrations were used as primary predictor variables.

The feeding route (oral vs. tube feeding), age at weaning, and presence of feeding difficulties such as increased work of breathing during feeding, diaphoresis, choking or coughing during feeding and inability to complete feeds, were noted from the infant's clinical records. The requirement for tube feeding was used as a surrogate marker for the severity of feeding difficulty. The levels of feeding difficulty were categorized as (1) no difficulty, (2) feeding difficulty but no need for tube feeding, and (3) feeding difficulty requiring tube feeding.

#### Postoperative Outcomes

Data were collected for length of stay in the pediatric intensive care unit (ICU) and in hospital, duration of mechanical ventilatory support, use of inotropic support (as measured by vasoactive inotrope score, VIS), need for extracorporeal membrane oxygenation (ECMO) support, delayed chest closure, and survival at 30 days, 6 months and 1 year after surgery. The occurrence of complications were also recorded, including sepsis or documented infections (e.g., culture-positive bacteremia, wound infection or dehiscence, urinary tract infection, pneumonia as defined by positive endotracheal tube aspirate with chest radiographic changes), low cardiac output state, episodes of reintubation, necrotizing enterocolitis, arrhythmias, renal impairment as defined by increase in serum creatinine >2 times upper limit of normal during the admission), and neurological morbidity (e.g., seizures, stroke, intracranial bleed).

The primary outcome variables were mortality at 6 and 12 months, and the presence of postoperative complications. Secondary outcomes variables included VIS, days on mechanical ventilation, days in ICU, and days in hospital.

#### Definition of Malnutrition

Malnutrition can be defined as “an imbalance between nutrient requirement and intake, resulting in cumulative deficits of energy, protein, or micronutrients that may negatively affect growth, development, and other relevant outcomes” (18). In this study, the WHO growth reference interpretation of cut-offs for malnutrition were used. Infants with *z-*scores lower than−2.0 in expected weight for age, or height for age were considered as malnourished (15).

### Statistical Analysis

Data were presented as mean with standard deviation (SD), or median with interquartile range, IQR (1^st^-3^rd^ quartile), or frequencies with percentages (%), as appropriate. Infants with no missing values in any predictor variables pre-surgery, outcome variables, and covariates were included in the complete analysis set. To address the potential bias due to missing values, missing values were replaced by multiple imputations (MI) by chained regression using all the aforementioned variables measured as predictors (19, 20). The MI procedure was performed with 20 sets of imputations.

The associations between the 6-month mortality, 12-month mortality, and postoperative complications with the preoperative nutritional status were analyzed by the logistic regression models. The associations between VIS, days on mechanical ventilation, days in ICU, and days in hospital with the preoperative nutritional status were analyzed using the median regression models. All the models were adjusted for potential covariates–sex, gestational age at birth, birth weight and length z-scores, cyanotic vs. acyanotic cardiac lesion, need for medications before surgery, need for tube feeding, age at surgery, priority of surgery, and RACHS-1 risk categories. The models also used cluster-standard errors to account for intra-cluster correlation among the same infants having multiple surgeries included in the analysis. The above associations were assessed in two sets of models. First, using the MI imputed analysis set, and second, using the complete analysis set. All analyses were performed in Stata/SE 16.1 for Windows (StataCorp, College Station, TX, USA). A *p*-value < 0.05 was interpreted as evidence of statistical significance.

## Results

### Study Population

**Figure 1:**
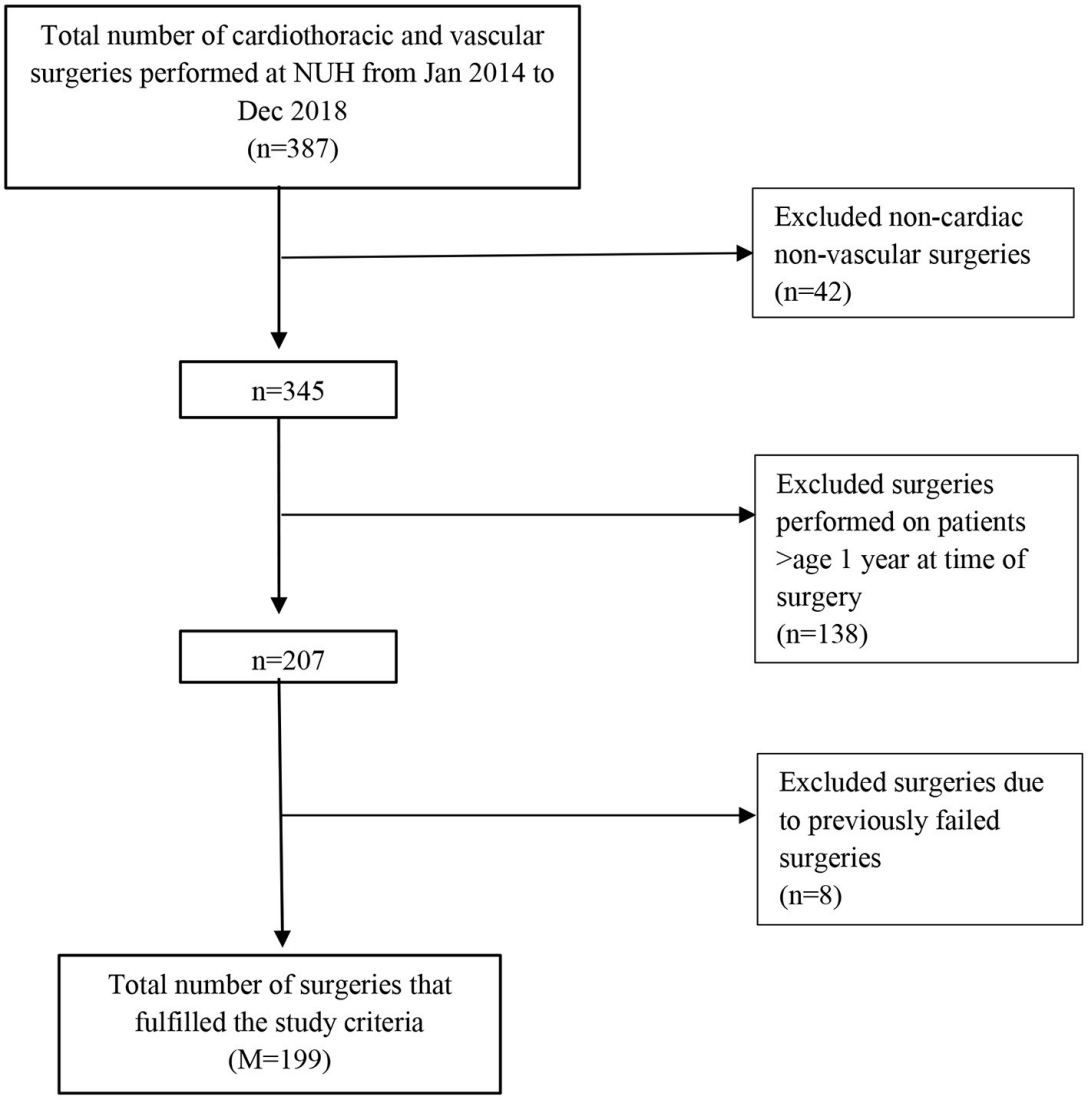
Study inclusion flowchart.

During the study period, 387 cardiac, thoracic and vascular surgical operations were conducted, of which 199 operations were eligible for inclusion. Non-cardiac, non-vascular surgeries (*n* = 42), as well as those performed on patients over age 1 year at time of surgery (*n* = 138) were excluded from the study. Operations that were a result of a previously failed operation were also excluded (*n* = 8) (Figure 1).

A total of 167 infants underwent 199 cardiac surgeries. More than 80% of the surgeries were in RACHS-1 categories 2 and 3. Forty two percent (83/199) of the surgeries were in RACHS-1 category 2, and 39.9% (79/199) in RACHS-1 category 3; 82.4% (164/199) of the operations were performed electively (Table 1). Table 2 showed the breakdown of the cohort into RACHS-1 categories 1, 2, 3, and 4-6, comparing their WAZ, LAZ, body mass index (BMI) and BMI-for-age z-score. These parameters were not significantly different across the RACHS-1 categories.

**Table 1:** Demographic and clinical characteristics^*^.

**Characteristic**	
**At birth characteristic (*****N*** **=** **167)**	
Sex	
Male	102 (61.1)
Female	65 (38.9)
Genetic syndrome	49 (29.3)
Gestational age at birth, weeks	38.0 ± 2.2
Preterm birth (<37 weeks)	30 (18.0)
Birth weight, kg	2.89 ± 0.61
Birth weight z-score	−0.29 ± 1.76
Birth length, cm	47.9 ± 3.5
Birth length z-score	−0.19 ± 1.41
**Pre-operative characteristic (*****M*** **=** **199)**	
Age at surgery, days	94 ± 95
Anthropometric data	
Pre-operative weight, kg	4.44 ± 1.80
Weight-for-age z-score	−1.31 ± 1.79
Weight-for-age z-score < -2.0	65 (32.7)
Pre-operative length, cm	56.1 ± 8.6
Length-for-age z-score	−0.64 ± 2.18
Length-for-age z-score < -2.0	42 (21.1)
Feeding	
Breast milk	56 (28.1)
Formula milk	54 (27.1)
Mixed feeding	89 (44.7)
Feeding difficulty	
No difficulty	91 (45.7)
Feeding difficulty but no need for tube feeding	65 (32.7)
Feeding difficulty requiring tube feeding	43 (21.6)
Referral to feeding team	71 (35.7)
Serum albumin, g/L	37.6 ± 7.1
Serum albumin <30 g/L	30 (15.13)
Hemoglobin, g/dL	12.5 ± 2.5
Pre-operative medications	
Diuretics	77 (38.7)
Prostaglandin E1	44 (22.1)
**Surgical Characteristic (*****M*** **=** **199)**	
Surgical priority	
Elective	164 (82.4)
Emergency	35 (17.6)
RACHS-1 risk category	
1	19 (9.6)
2	83 (41.9)
3	79 (39.9)
4	14 (7.1)
≥5	3 (1.5)
CPB time, min [median (IQR)]	70 (0–121)
AXC time, min [median (IQR)]	42 (30–75)
DHCA time, min [median (IQR)]	14 (7–33)
Delayed sternal closure, days	32 (16.1)

Values represent mean ± SD, standard deviation; or frequency (percentage), unless specified otherwise. ^*^199 cardiac surgeries (M) were performed on 167 infants (N). AXC, aortic cross-clamp; CPB, cardiopulmonary bypass; DHCA, deep hypothermic circulatory arrest; RACHS-1, Risk Adjustment for Congenital Heart Surgery.

**Table 2:** Summary of preoperative anthropometric parameters by Risk Adjustment for Congenital Heart Surgery (RACHS)−1 risk categories.

**Preoperative anthropometric parameters**	**RACHS-1 risk categories, mean (SD)**				***p*-value**
	**1**	**2**	**3**	**4-6**	
	***n* = 19**	***n* = 83**	***n* = 79**	***n* = 18**	
Weight-for-age z-score	−2.07 (2.11)	−1.26 (1.52)	−1.16 (1.92)	−1.24 (1.83)	0.25
Length-for-age z-score	−0.72 (1.46)	−0.32 (2.05)	−0.94 (2.50)	−0.69 (1.63)	0.36
Body mass index (BMI) (kg/m^2^)	13.57 (2.62)	13.82 (2.14)	13.95 (3.75)	12.94 (2.38)	0.69
BMI-for-age z-score	−1.64 (1.68)	−1.53 (1.34)	−0.75 (2.62)	−1.14 (1.94)	0.086

*p-values are based on the Analysis of Variance (ANOVA)*.

Mean age at cardiac surgery was 94 days (SD 95). More than half (61.0%; 102/167) of the infants were male. Mean gestational age at birth was 38.0 weeks (SD 2.2) (range 26 to 41 weeks). Thirty infants (18.0%; 30/167) were born prematurely (<37 weeks gestation). Fifty-four percent (53.9%; 90/167) had cyanotic cardiac lesions. The commonest acyanotic and cyanotic lesions in our study population were ventricular septal defect (26.3%; 44/167), and tetralogy of Fallot (13.8%; 23/167), respectively (Table 3).

**Table 3:** Types of congenital heart disease (*N* = 167).

**Cardiac lesion**	***n* (%)**
**Cyanotic**	90 (53.9)
Tetralogy of Fallot	23 (13.8)
Transposition of great arteries	20 (12.0)
Total anomalous pulmonary venous return	8 (4.8)
Pulmonary atresia with intact ventricular septum	8 (4.8)
Hypoplastic left heart syndrome	6 (3.6)
Pulmonary atresia with ventricular septal defect	6 (3.6)
Double-outlet right ventricle (Fallot-type)	6 (3.6)
Double outlet right ventricle	5 (3.0)
Truncus arteriosus	3 (1.8)
Tricuspid atresia	2 (1.2)
Hypoplastic right heart syndrome	1 (0.6)
Other single ventricle type	2 (1.2)
**Acyanotic**	77 (46.1)
Ventricular septal defect	44 (26.3)
Patent ductus arteriosus	11 (6.6)
Aortic coarctation	6 (3.6)
Atrioventricular septal defect	5 (3.0)
Ventricular septal defect + aortic coarctation	5 (3.0)
Partial anomalous pulmonary venous return	1 (0.6)
Ebstein anomaly	1 (0.6)
Others e.g., double aortic arch	4 (2.4)

### Preoperative Characteristics

Mean preoperative WAZ and LAZ were−1.31 (SD 1.79), and−0.64 (SD 2.18), respectively. Mean preoperative serum albumin level was 37.6 g/L (SD 7.1); 15.1% (30/199) had low serum albumin (<30 g/L). The mean preoperative Hb level was 12.5 g/dL (SD 2.5) (Table 1).

Infants encountered feeding difficulties, including but not limited to increased work of breathing, diaphoresis, choking or coughing during feeding, and inability to complete feeds in 54.3% (108/199) of the operations before surgery, of which 35.7% (71/199) required the infant to be referred to the feeding team before surgery. For 21.6% (43/199) of the surgeries, the infants were tube-fed preoperatively.

### Postoperative Outcomes

The median duration of ICU and hospital stay were 9 (IQR 6–19) days, and 17 (IQR 10–36) days, respectively. The median duration of mechanical ventilation was 5 (IQR 3–8) days, and median VIS was 7.5 (IQR 2.0–10.8). The postoperative complication rate was 63.3% (126/199). The types of postoperative complications were summarized in Table 4, the commonest being prolonged stay in the ICU of >14 days (29.1%; 58/199) and acute kidney injury (19.1%; 38/199).

**Table 4:** Postoperative complications (*M* = 199).

**Complications**	***n* (%)**
Intensive care unit stay >14 days	58 (29.1)
Acute kidney injury	38 (19.1)
Nosocomial infection except wound infection	28 (14.1)
Arrhythmia/ conduction disorders	23 (11.6)
Chylothorax	13 (6.5)
Wound infection	13 (6.5)
Complications related to instrumentation/ catheters/ devices	12 (6.0)
Unplanned reoperation	11 (5.5)
Diaphragmatic palsy	9 (4.5)
Cardiopulmonary resuscitation	7 (3.5)
Postoperative extracorporeal membrane oxygenation	6 (3.0)
Reintubation within 24 h	6 (3.0)
Venous thrombosis	4 (2.0)
Chest re-exploration	4 (2.0)
Seizures/ strokes	3 (1.5)
Vocal cord palsy/ subglottic stenosis	2 (1.0)
Arterial thrombosis	2 (1.0)
Visit to emergency department/ Readmission	2 (1.0)

There were only 2 deaths in the first 30 days post-surgery. One of the infants died from an underlying primary metabolic disorder, while the second infant's death was attributed to the underlying cardiac lesion, complicated by pulmonary hypertension. The 6 and 12-month all-cause mortality rates were 4.5% (9/199), and 6.5% (13/199), respectively (Table 5). The median age of death was 6.0 (IQR 3.0–9.3) months, and median time to death was 100 (IQR 60–269) days after surgery. The commonest cause of death was sepsis (56.3%; 9/16). Other causes included pulmonary hypertension (31.3%, 5/16) and necrotizing enterocolitis (6.3%; 1/16).

**Table 5:** Surgical outcomes (*M* = 199).

**Outcome**	
Mortality	
6-month mortality	9 (4.5)
12-month mortality	13 (6.5)
Overall mortality	16 (8.0)
Postoperative complication	126 (63.3)
Vasoactive inotrope score	7.5 (2.0–10.8)
Days on mechanical ventilation	5 (3–8)
Days in intensive care unit	9 (6–19)
Days in hospital	17 (10–36)

*Values represent median (IQR); or frequency (percentage)*.

### Association Between Preoperative Anthropometry and Postoperative Outcomes

There were some differences in covariates and outcomes among infants included in the complete analysis set, and those with some missing values (Supplementary Table 1). For example, the complete analysis set had higher gestational age, preoperative WAZ and LAZ, as well as lower mortality at 6 and 12 months. Therefore, the analysis based on the multiple imputation set (without excluding any infants and replacing missing values with plausible values) was considered as the primary analysis, and the analysis based on the complete analysis set as a sensitivity analysis.

Results from regression models based on the multiple imputation analysis and complete analysis sets, were summarized in Tables 6, 7, respectively.

**Table 6:** Summary of regression models for association between preoperative nutritional status and surgery outcomes based on the multiple imputation analysis set.

**Preoperative**
**nutritional**	**Odds ratio (** ***p*** **-value)**			**Difference in medians (** ***p*** **-value)**			
**status**	**[95% confidence interval]** ^**a**^			**[95% confidence interval]** ^**b**^			
	**6-month**	**12-month**	**Postoperative**	**Vasoactive**	**Days on**	**Days in**	**Days in**
	**mortality**	**mortality**	**complications**	**inotrope score**	**mechanical ventilation**	**intensive care unit**	**hospital**
	**(M/N = 199/178)**	**(M/N = 199/178)**	**(M/N = 199/178)**	**(M/N = 199/178)**	**(M/N = 199/178)**	**(M/N = 199/178)**	**(M/N = 199/178)**
Weight-for-age	0.42 (0.008)**	0.75 (0.32)	0.55 (0.02)**	0.43 (0.34)	−0.27 (0.38)	−0.56 (0.35)	−1.68 (0.36)
z score	[0.22, 0.79]	[0.42, 1.32]	[0.33, 0.93]	[-0.45, 1.31]	[-0.88, 0.33]	[-1.74, 0.62]	[-5.31, 1.96]
Length-for-age	1.11 (0.58)	1.02 (0.89)	1.26 (0.12)	−0.26 (0.55)	0.161 (0.494)	−0.58 (0.43)	−1.76 (0.43)
z score	[0.76, 1.63]	[0.73, 1.44]	[0.95, 1.67]	[-1.10, 0.59]	[-0.30, 0.63]	[-2.03, 0.87]	[-6.17, 2.64]
Hemoglobin	1.11 (0.52)	1.06 (0.64)	1.05 (0.58)	0.31 (0.15)	0.01 (0.95)	−0.25 (0.45)	−0.77 (0.25)
(g/dL)	[0.82, 1.49]	[0.83, 1.34]	[0.89, 1.24]	[-0.11, 0.74]	[-0.29, 0.32]	[-0.90, 0.40]	[-2.09, 0.55]
Serum albumin	1.49 (0.71)	1.553 (0.67)	0.71 (0.59)	−2.11 (0.06)	0.83 (0.32)	−0.07 (0.98)	−0.02 (0.99)
level <30 (g/L)	[0.18, 12.82]	[0.21, 11.74]	[0.21, 2.44]	[-4.28, 0.06]	[-0.82, 2.48]	[-4.86, 4.72]	[-9.86, 9.81]

M, Number of operations; N, Number of patients. ^a^Based on the Logistic Regression With Robust Clustered-Standard Error and Adjusted for Covariates. ^b^Based on the Median Regression With Robust Clustered-Standard Error and Adjusted for Covariates. All models were adjusted for covariates-gender, gestational age, age at surgery, lesion type, feeding type, level of difficulty in feeding, referral to feeding team, risk adjustment classification for congenital heart surgery, cardiopulmonary bypass time, and aortic clamp time. ^**^ p-value < 0.05.

**Table 7:** Summary of regression models for association between preoperative nutritional status and surgery outcomes based on the complete analysis set.

**Preoperative**	**Odds ratio (** ***p*** **-value)**				**Difference in medians (** ***p*** **-value)**			
**nutritional status**	**[95% confidence interval]** ^**a**^				**[95% confidence interval]** ^**b**^			
	**6-month**	**12-month**	**Postoperative**	**Vasoactive**	**Days on**	**Days in**	**Days in**
	**mortality**	**mortality**	**complications**	**inotrope score**	**mechanical ventilation**	**intensive care unit**	**hospital**
	**(M/N = 165/150)**	**(M/N = 165/150)**	**(M/N = 165/150)**	**(M/N = 165/150)**	**(M/N = 165/150)**	**(M/N = 165/150)**	**(M/N = 165/150)**
Weight-for-age	0.58 (0.02)**	0.80 (0.33)	0.49 (0.03)**	0.44 (0.42)	−0.26 (0.42)	−0.32 (0.69)	−1.74 (0.36)
z score	[0.37, 0.91]	[0.51, 1.26]	[0.26, 0.95]	[-0.64, 1.53]	[-0.89, 0.38]	[-1.89, 1.26]	[-5.52, 2.03]
Length-for-age	1.10 (0.52)	1.02 (0.91)	1.27 (0.19)	0.004 (0.99)	0.22 (0.21)	−0.51 (0.52)	0.89 (0.54)
z score	[0.82, 1.48]	[0.72, 1.44]	[0.89, 1.81]	[-0.84, 0.84]	[-0.13, 0.56]	[-2.08, 1.06]	[-1.99, 3.78]
Hemoglobin (g/dL)	1.16 (0.49)	1.14 (0.48)	1.09 (0.49)	0.30 (0.28)	0.17 (0.24)	−0.36 (0.40)	−0.42 (0.62)
	[0.77, 1.74]	[0.79, 1.63]	[0.86, 1.37]	[-0.25, 0.85]	[-0.11, 0.45]	[-1.21, 0.49]	[-2.05, 1.22]
Serum albumin level	0.99 (0.99)	1.07 (0.95)	1.67 (0.47)	−2.02 (0.07)	1.61 (0.05)**	0.86 (0.69)	3.00 (0.58)
<30 (g/L)	[0.06, 15.78]	[0.12, 9.53]	[0.41, 6.73]	[-4.15, 0.12]	[0.04, 3.19]	[-3.48, 5.21]	[-7.69, 13.69]

M, Number of operations; N, Number of patients. ^a^Based on the Logistic Regression With Robust Clustered-Standard Error and Adjusted for Covariates. ^b^Based on the Median Regression With Robust Clustered-Standard Error and Adjusted for Covariates. All models were adjusted for covariates-gender, gestational age, age at surgery, lesion type, feeding type, level of difficulty in feeding, referral to feeding team, risk adjustment classification for congenital heart surgery, cardiopulmonary bypass time, and aortic clamp time. ^**^ p-value < 0.05.

#### 6- and 12-Month Mortality

The logistic regression model based on the MI analysis set showed statistically significant association between preoperative WAZ and 6-month mortality (*p* = 0.008). The odds ratio for 6-month mortality for a 1-unit decrease in WAZ in the model was 2.38 [95% confidence interval (CI) 1.26–4.54]. Similarly, the preoperative the decrease in WAZ was associated with increase in the 12-month mortality, but this association was not statistically significant (Table 6). Results based on the complete analysis set were similar to the multiple imputation set (Table 7).

#### Postoperative Complications

In line with the mortality outcomes, the logistic regression model based on the multiple imputation set showed statistically significant association between preoperative WAZ and postoperative complications (*p* = 0.02). A 1-unit decrease in WAZ was associated with 1.82 OR (95% CI 1.08–3.03) (Table 6). Similar result was observed based on the complete set analysis (Table 7).

#### Secondary Outcomes

There was no statistically significant association between VIS, days on mechanical ventilation, days in ICU, and days in hospital with any of the nutritional predictors based on the multiple imputation set as well as the complete analysis set, except between serum albumin level <30 g/L and days on mechanical ventilation based on the complete analysis set (odds ratio 1.61; *p* = 0.05) which was non-significant based on the multiple imputation set.

## Discussion

In this study, we demonstrated that 1 in 3 infants presenting for congenital heart surgery were malnourished with preoperative weight-for-age z-score (WAZ) < −2.0. Preoperative feeding difficulties were present in more than half of the surgeries, of which 21.6% required the infants to be tube fed. Our analysis revealed that lower preoperative WAZ is significantly associated with increased 6-month mortality after CHD surgery, and postoperative complications. As mentioned, our study is unique as it focuses specifically on infants who may be particularly vulnerable to nutritional deficits. However, owing to the retrospective nature of our study, the causal relationship between malnutrition and disease severity, and therefore, with surgical outcomes could not be ascertained.

A significant quandary in diagnosing malnutrition is a lack of uniformity in definition of the term (21). Oftentimes, nutritional status in infants is characterized in terms of anthropometric indices comparing weight and height to population norms. In the United States, a study of hospitalized children with heart disease reported 33% prevalence of acute malnutrition, defined as the ratio of the child's weight to the mean weight-for-height <0.89, and 64% chronic malnutrition, defined as the ratio of the child's height to mean height-for-age <0.94 (22). In French infants and young children with CHD, the incidence of malnutrition, defined as weight-for-height ratio <0.80, was 15% (6). In developing countries, this incidence is even higher (5, 13). The WHO defines malnutrition as WAZ or LAZ below−2.0 (23, 24). The term *underweight* is used to describe children with low WAZ, whereas low LAZ is thought to represent *stunting*, a chronically malnourished state with diminished somatic growth (25). The occurrence of low WAZ and LAZ demonstrated in our study (32.7 and 21.1%, respectively) indicates that low anthropometric indices are common in infants with CHD even in relatively resource-rich Singapore. This is similar to the findings of a recent analysis of the Society of Thoracic Surgeons Congenital Heart Surgery Database (STS-CHSD), which encompasses approximately 98% of all pediatric cardiac operations performed annually in the United States. The STS-CHSD analysis demonstrated that, in infants 1 month to 1 year of age, 41.37% and 36.64% had WAZ < -2, and LAZ < -2, respectively (26). Therefore, if low anthropometric indices are found to be a modifiable risk factor for poor surgical outcomes, there is a significant proportion of infants with CHD who would benefit from optimization of nutrition.

Several studies of children with CHD demonstrated an association between poor nutritional status and worse outcomes after congenital heart surgery. A retrospective study of children aged 0 to 5 years who underwent cardiac surgery at the Seattle Children's Hospital found that for those with preoperative WAZ ≤ −2.0, each unit reduction of preoperative WAZ was associated with a 2.1% increased risk of mortality, 0.7% increased risk of cardiac arrest, 0.8% increased risk of infection, an average of 1.9 additional hours of mechanical ventilation, and 5.3 additional hours of ICU stay (26, 27). Lim and colleagues reported that preoperative WAZ ≤ - 2.0 was associated with higher 30-day mortality following CHD surgery in infants and children under the age of 10 years (28). In comparison, our 30-day mortality was a relatively low-frequency event; we demonstrated that lower preoperative WAZ is significantly associated with increased 6-month mortality after CHD surgery (OR 2.38, 95% CI 1.26–4.54; *p* = 0.008), and postoperative complications (OR 1.82, 95% CI 1.08–3.03; *p* = 0.02). This is in keeping with the STS-CHSD analysis which showed that lower values for WAZ are significantly associated with increased mortality, and composite outcome of mortality or major complication (26). Mitting and colleagues found that low WAZ was associated with increased duration of mechanical ventilation and mortality in neonates undergoing surgery for CHD (29). Lower WAZ has also been associated with increased hospital length of stay after bidirectional cavopulmonary connection surgery, as well as increased risk of infection, increased in-hospital mortality, and increased length of stay after Fontan operation (30–32).

Intuitively, one may attribute the association between low WAZ and increased mortality to the fact that malnutrition itself contributes to postoperative complications. This is entirely reasonable because malnutrition is known to result in immune impairment, and may contribute to respiratory muscle weakness, cardiovascular derangements, and impaired wound healing (33–35). Another possible explanation is that low WAZ may be a reflection of the severity of cardiac disease in which infants with more severe disease are more likely to have poorer surgical outcome, and are more likely to suffer from malnutrition due to increased metabolic rate, poor nutrient absorption, and/or feeding difficulty. Although these data cannot conclude that improved nutrition would prevent adverse surgical outcomes, the associations shown in this study suggest a potential role for nutritional intervention in this population.A recent randomized controlled trial in malnourished infants with CHD demonstrated improved outcomes with perioperative nutritional prehabilitation prior to elective CHD surgery. The investigators showed that a 2-week prehabilitation program was associated with better anthropometric measurements (WAZ, HAZ, and body mass index), shorter duration of mechanical ventilation, and shorter ICU and hospital stay postoperatively (36).

Although nutritional status in infants is often described in terms of anthropometric indices, one would be cognizant that weight-based index may be affected by fluid shifts, and can be especially confounded in the infant with heart disease. As anthropometric indices may not always specifically reflect nutritional status, other measures of nutrition such as biochemical markers have been employed to clarify the role of nutrition in preoperative assessment and optimization. Among the biochemical parameters, serum albumin is commonly measured to assess nutritional status. In our study, although hypoalbuminemia (<30 g/L) was present in 15% of the infants, preoperative serum albumin was not associated with surgical outcomes. Despite its historical popularity, studies are inconsistent for proving the utility of serum markers as determinants of patient's nutritional status. A low preoperative serum albumin (<30 g/L) was associated with increased postoperative infections and mortality among children undergoing cardiac surgery (37, 38). Using serum albumin level as a marker of chronic malnourishment, it has been found that, after adjusting for RACHS-1 risk categories, a higher albumin level was associated with a decrease in B-type natriuretic peptide (BNP), and an overall trend toward decreased duration of dopamine requirement (33). However, in a case-control observational study of children aged 3 to 92 months with uncorrected symptomatic CHD, serum proteins and albumin were similar in cases and healthy controls, and in the acyanotic and cyanotic groups (39). The major consensus in the literature is that these laboratory markers are not reliable by themselves. This is because serum albumin is characterized as a negative acute-phase protein, and its pool is affected by a number of inflammatory conditions and drugs. Furthermore, its long half-life of 3 weeks, wide distribution, reduced degradation during low protein intake, and frequent supplementation after cardiac surgery further limit its utility as a marker (40). Therefore, other anthropometric measurements, such as measurement of mid-arm circumference (which would not be affected by fluid shifts), could be explored as a markers of a child's nutritional status.

As preoperative WAZ has a significant impact on post-surgical outcomes for infants undergoing cardiac surgery, close attention should be paid to it, especially since it is readily measurable. Our findings suggest that delaying elective surgery to optimize preoperative WAZ may be beneficial. However, further risk-benefit assessment based on illness severity will be needed to determine whether such a delay can be safely considered for all infants with CHD. In any case, as preoperative WAZ improves outcomes in terms of postoperative complications and 6-month mortality, we recommend that all infants be referred to optimize preoperative WAZ using a consensus-based standardized nutritional pathway before surgery. A clinical nutrition study by Marino and colleagues showed that such a preoperative pathway for CHD infants, which involved a dietician contacting parents of infants with the highest risk of growth failure weekly, reviewing weight gain and providing feeding support, was associated with improved growth, and reduced durations of mechanical ventilation and ICU stay (41).

### Study Limitations

Our study has a few limitations. First, our analysis was based on a single-center retrospective database. Nevertheless, the study center is the second largest public health institution in Singapore with patients treated from all over nation; hence our study population can be considered to be a representative sample of the country. If all things else being equal, this study can be generalized as the findings are consistent with other published data. Secondly, the anthropometric indices used here represent a single time point; trends in anthropometric indices over time may be more predictive of outcomes than isolated measurements. Thirdly, infants with CHD may have associated disorders of pulmonary, digestive, musculoskeletal and other organ systems, which could contribute to feeding difficulty, failure to thrive, and poorer postoperative outcomes, thus confounding the study. However, as their presentations were too varied to be categorized meaningfully, an all-comer approach was adopted, instead of excluding infants with comorbidities, or including those with selected comorbidities. Lastly, the study was not powered for multiple hypothesis testing and we do not rule out the possibility of inflated type-I error (false positive). Nevertheless, this may not detract from the finding that poor feeding and malnutrition in terms of low preoperative WAZ portend a poorer surgical outcome in infants with CHD. Some of the statistically non-significant associations between the nutritional status and surgical outcomes could be related to insufficient statistical power.

## Conclusion

We demonstrated that low preoperative weight-for-age z-score was associated with increased risk of postoperative complications and 6-month mortality in infants with CHD after cardiac surgery. We recommend that close attention be paid to the preoperative weight-for-age z-score, and that all infants be referred for assessment and optimization of their nutritional status prior to CHD surgery. Implementation of pre- and postoperative nutritional protocols for infants with CHD can standardize feeding practices and improve outcomes.

## Data Availability Statement

The raw data supporting the conclusions of this article will be made available by the authors, without undue reservation.

## Ethics Statement

This was a single-center, retrospective cohort study that was reviewed and approved by the Domain Specific Review Board (DSRB), National Healthcare Group, Singapore, with a waiver of informed consent.

## Author Contributions

JL and RW participated in data collection, data analysis, statistical analysis, and writing of the manuscript. MG participated in statistical analysis, reviewed, and approved the final manuscript. YL participated in performance of the research, reviewed, and approved the final manuscript. LT participated in data analysis and approved the final manuscript. SQ participated in performance of the research and approved the final manuscript. MA participated in conceptualization, performance of the research, reviewed the data, and approved the final manuscript. CC acted as the senior author, designed the study, reviewed the analyzed data, reviewed and revised the manuscript, and approved the final manuscript. All authors contributed to the article and approved the submitted version.

## Funding

CC is supported by the National Medical Research Council, Singapore (NMRC/CNIG19nov-0006).

## Conflict of Interest

The authors declare that the research was conducted in the absence of any commercial or financial relationships that could be construed as a potential conflict of interest.

## Publisher's Note

All claims expressed in this article are solely those of the authors and do not necessarily represent those of their affiliated organizations, or those of the publisher, the editors and the reviewers. Any product that may be evaluated in this article, or claim that may be made by its manufacturer, is not guaranteed or endorsed by the publisher.

## Abbreviations

AXC: aortic cross-clamp
CHD: congenital heart disease
CI: confidence interval
CPB: cardiopulmonary bypass
DHCA: deep hypothermic circulatory arrest
Hb: hemoglobin
ICU: intensive care unit
IQR: interquartile range
LAZ: length-for-age z-score
LOS: length of stay
RACHS: risk adjustment for congenital heart surgery
SD: standard deviation
VIS: vasoactive inotrope score
WAZ: weight-for-age z-score
WHO: world health organization.
