# Supplementary material for: The Associations Between Preoperative Anthropometry and Postoperative Outcomes in Infants Undergoing Congenital Heart Surgery
Source: Front Cardiovasc Med. 2022 Apr 1;9:812680. doi: 10.3389/fcvm.2022.812680 (PMC9010609; doi:10.3389/fcvm.2022.812680)
Supplement: Supplementary file 1 [file Table_1.docx]

**SUPPLEMENTARY MATERIALS**

**SUPPLEMENTARY TABLE 1.** Comparison of covariates and surgical outcomes in the complete analysis set vs. incomplete analysis set

|  |  | **Complete analysis set** | **Incomplete analysis set** |  |
| --- | --- | --- | --- | --- |
| At birth characteristics | Statistics | N = 150 | N = 32* | p-value |
| Female | n (%) | 57 (38.0) | 15 (46.9) | 0.426 |
| Gestational age (weeks) | Mean (SD) | 38.1 (1.74) | 36.8 (3.72) | 0.002 |
| At pre-operation characteristics | Statistics | M = 165 | M = 34 | p-value |
| Age (days) | Mean (SD) | 97.1 (98.49) | 77.1 (77.08) | 0.266 |
| Weight-for-age z score | Mean (SD) | -1.08 (1.58) | -2.41 (2.30) | <0.001 |
| Length-for-age z score | Mean (SD) | -0.45 (2.00) | -1.93 (2.89) | 0.002 |
| Hemoglobin (g/dL) | Mean (SD) | 12.65 (2.43) | 11.78 (2.59) | 0.060 |
| Serum albumin <30 g/L | n (%) | 27 (16.4) | 3 (15.8) | >0.999 |
| Feeding type | n (%) |  |  | 0.914 |
| Breast milk |  | 46 (27.9) | 10 (29.4) |  |
| Formula milk |  | 46 (27.9) | 8 (23.5) |  |
| Mixed feeding |  | 73 (44.2) | 16 (47.1) |  |
| Feeding difficulty | n (%) |  |  | 0.629 |
| No difficulty |  | 75 (45.5) | 16 (47.1) |  |
| Have difficulty but no tube feeding |  | 56 (33.9) | 9 (26.5) |  |
| Have difficulty and require tube feeding |  | 34 (20.6) | 9 (26.5) |  |
| Referral to feeding team | n (%) | 56 (33.9) | 15 (44.1) | 0.326 |
| Lesion type | n (%) |  |  | 0.575 |
| Cyanotic |  | 96 (58.2) | 18 (52.9) |  |
| Acyanotic |  | 69 (41.8) | 16 (47.1) |  |
| RACHS-1 risk category | n (%) |  |  | 0.008 |
| 1 |  | 12 (7.3) | 7 (21.2) |  |
| 2 |  | 74 (44.9) | 9 (27.3) |  |
| 3 |  | 68 (41.2) | 11 (33.3) |  |
| ≥4 |  | 11 (6.7) | 6 (18.2) |  |
| CPB time (minute) | Median (IQR) | 73 (121.0) | 0 (127.0) | 0.023 |
| AXC time (minute) | Median (IQR) | 28 (59.0) | 0 (59.0) | 0.108 |
| Surgical outcomes | Statistics | M = 165 | M = 34 | p-value |
| 6-month mortality  12-month mortality  Postoperative complication  Vasoactive inotrope score | n (%)  n (%)  n (%)  Median (IQR) | 7 (4.2)  9 (5.4)  104 (63.0)  7.5 (8.5) | 6 (17.7)  7 (20.6)  22 (64.7)  5 (9.0) | 0.011  0.008  0.509  0.067 |
| Days on mechanical ventilation | Median (IQR) | 5 (4.0) | 5.5 (8.5) | 0.334 |
| Days in intensive care unit | Median (IQR) | 9 (12.0) | 9.5 (23.0) | 0.695 |
| Days in hospital | Median (IQR) | 15 (22.0) | 38 (74.0) | 0.024 |

N, Number of patients; M, Number of surgeries; n, frequency; IQR, Interquartile range; RACHS-1, Risk Adjustment for Congenital Heart Surgery; CPB, Cardiopulmonary bypass; AXC, Aortic cross-clamp.

*Four subjects with two surgeries were counted in both the complete analysis set and the incomplete analysis set as data related to one surgery was complete but not for another surgery.
